# Consensus about managing gastrointestinal and cardiovascular risks of nonsteroidal anti-inflammatory drugs?

**DOI:** 10.1186/s12916-015-0291-x

**Published:** 2015-03-19

**Authors:** Neville D Yeomans

**Affiliations:** School of Medicine, University of Western Sydney, Locked Bag 1797, Penrith, NSW 2751 Australia; Office for Research, Austin Health, Heidelberg, Vic 3084 Australia

**Keywords:** Cardiovascular risk, COX-2 selective inhibitors, Gastrointestinal hemorrhage, Gastroprotection, Non-steroidal anti-inflammatory drugs, Peptic ulcer

## Abstract

In a recently published article in *BMC Medicine*, Scarpignato and colleagues present the results of a consensus conference that addressed several aspects of the management of pain in patients with osteoarthritis. The main areas covered include the relative safety in regard to gastrointestinal and cardiovascular adverse events of non-selective ‘traditional’ non-steroidal anti-inflammatory drugs (NSAIDs) versus cyclooxygenase-2 selective NSAIDs. The role of co-therapy with proton pump inhibitors in enhancing gastrointestinal safety is also reviewed.

This commentary focuses on two areas that the consensus conference addressed, i) the whole length of gastrointestinal tract risk profile of the various NSAIDs (not just the ulcer risks in stomach and duodenum); ii) more recent information, but still some uncertainties, about the cardiovascular risks associated with the two classes of NSAID in general, and naproxen in particular.

Please see related article: http://dx.doi.org/10.1186/s12916-015-0285-8

## Background

As life expectancy in many countries increases into the 80s and beyond, degenerative joint disease is creating an increasing burden for patients and healthcare systems. For osteoarthritis especially, non-steroidal anti-inflammatory agents (NSAID) remain the most effective option for pain relief, short of surgical alternatives such as joint replacement [1]. However, gastrointestinal (GI) ulcers and their complications are well-known NSAID side effects that are more prevalent in the elderly and are, at times, life-threatening [2].

The recognition that NSAIDS damage the stomach and duodenum (at least partly) by blocking the mucosal production of protective prostaglandins catalyzed by cyclooxygenase (COX)-1 [3] led to the development of COX-1-sparing NSAIDs. These selectively inhibit COX-2, which mediates synthesis of pro-inflammatory prostaglandins. The strategy has been successful: highly selective COX-2 inhibitors do reduce (but do not eliminate) the risk of GI ulceration [4,5]. However, an unanticipated risk that surfaced in several randomized studies was an increase in adverse cardiovascular (CV) events in patients taking COX-2 inhibitors for months or years [6-8]. The European Medicines Agency responded promptly, stating, in 2005, that ‘*COX-2 inhibitors must not be used in patients with established ischaemic heart disease and/or cerebrovascular disease* …’ [9]. On the other hand, the US Food and Drug Agency (FDA), in the same year, declined to make such a limiting statement – noting that it was unclear whether COX-2 inhibitors carried a greater vascular risk than the older non-selective NSAIDs (nsNSAIDs), that further research was required, and that in the meantime warnings about the possibility of increased CV risk with all NSAIDs should be included in drug labeling [10].

Thus, clinicians and their patients face some dilemmas about how to balance the GI and CV risks, especially in patients known to be at increased risk for both, as occurs in many elderly patients. Cryer, in a submission on behalf of consumers to a 2014 FDA hearing that was contemplating a labeling change based solely on CV risk, emphasized the need for a comprehensive risk assessment: ‘*In the process of addressing cardiovascular health in the setting of NSAID use, which we applaud, we* […] *would not want you to inadvertently increase the risk of other untoward outcomes associated with NSAIDs, such as GI and renal toxicities*’ [11].

It is timely to review the area; thus, in a recent article published in *BMC Medicine*, Scarpignato et al. [12] report on a recent consensus meeting that has updated earlier guidelines using more recent information.

## Discussion

Scarpignato et al. [12] used a modified Delphi approach to gauge levels of agreement and opinions on the level of evidence for nine statements about various aspects of NSAID use. These ranged from efficacy for pain relief, a comparison of GI risks with different NSAIDs, to a comparison of the CV risks with different NSAIDS. The panel was an international multidisciplinary group. It is perhaps a pity that their meeting was now more than three years ago, but the authors updated their literature search in the interval for this publication. The result is a helpful distillation of expert opinion on the areas covered. This commentary will focus on two aspects. Firstly, the consensus statement looks more comprehensively than others at the GI risks of NSAIDS from top to bottom of the GI tract. While the life threatening complications of NSAIDS (including low-dose aspirin) arise mainly from ulcers in the stomach and duodenum, it is increasingly recognized that small intestinal ulceration is also common as one cause of iron-deficiency anemia in NSAID users and, occasionally, of frank GI hemorrhage. Looking at GI risk in its totality, statement 4 from the consensus conference reads in part: ‘*NSAID use is associated with increased risk of adverse events throughout the entire GI tract*.’ The levels of agreement and of supporting evidence were both high. There is good evidence, summarized in the consensus paper, that proton pump inhibitors (PPI) substantially reduce the risk of upper GI ulceration and complications of both nsNSAIDs and COX-2 inhibitors. However, it is not surprising that current evidence (rated level B by the conference) indicates that PPIs do not protect against ulceration in the near neutral pH milieu of the small intestine and colon.

The second issue worthy of comment is the conclusion the consensus group reached about whether some NSAIDs are safer than others from the standpoint of CV risk. Statement 8 reads: ‘*The risk of CV events associated with celecoxib use is similar to that associated with the use of most ns-NSAIDs*.’ Eighty-four percent of the panel agreed strongly or moderately, although only just over half the panel rated the level of evidence as high. They did not endorse earlier strong recommendations from bodies such as the American Heart Association and American College of Gastroenterology that naproxen should be the NSAID of choice for patients with high CV risk [13-16]. Instead, the treatment-guidance algorithm they propose allows either naproxen or low-dose celecoxib as the preferred agents in patients with high CV risk, adding in a PPI to either if patients are judged to also be at high GI risk.

As Scarpignato et al. [12] indicate, the evidence about whether naproxen has a lower CV risk has been conflicting; there is some pharmacokinetic basis to suspect it might. Aspirin exerts its prolonged anti-platelet effect because it irreversibly acetylates platelet cyclooxygenase [17]. However, other nsNSAIDs are reversible inhibitors of the enzyme, so their platelet inhibitory effect disappears as their plasma levels dissipate [18]. Naproxen is one of the longer acting nsNSAIDs, with a plasma elimination half-life of about 14 hours [19]; a small study of volunteers given a single dose of 1,000 mg found platelet aggregation still reduced after 24 hours in 60% of cases [20]. Thus, it is plausible that twice daily dosing may offer some protection against thrombotic events.

Seemingly in support of this, a recent large meta-analysis (the CNT collaboration) found that a coxib, diclofenac, or ibuprofen increased the rate of major vascular events by about a third (not quite significant for ibuprofen), but naproxen did not [21]. However, a meta-analysis is only as strong as its component parts, and a particular weakness of the CNT meta-analysis was that it had to indirectly compare the effects of the different drugs. That is to say, studies of drug A versus drug B were combined with studies of drug A versus placebo to estimate relative risks for drug B versus placebo.

As Scarpignato et al. [12] note, and a recent FDA hearing concluded, the best evidence should come from a large randomized controlled trial in arthritis patients at high CV risk; such a study is now nearing completion [22]. The Prospective Randomized Evaluation of Celecoxib Integrated Safety versus Ibuprofen Or Naproxen (PRECISION) trial, which was mandated by the FDA, has recruited more than 20,000 such patients. The last patient follow-up is scheduled for the end of 2015, so results can be anticipated during 2016 [23]. This study should give useful, real-world information to increase the evidence base for managing the high CV risk arthritis patient.

## Conclusions

Scarpignato et al. [12] have produced a valuable summary of the current state of knowledge about the GI and CV risks of both nsNSAIDs and COX-2 selective drugs, which will be helpful for clinicians managing patients with osteoarthritis. As they emphasize, there are still uncertainties regarding CV risk profiles of commonly used NSAIDs, and results of some ongoing research directed at this are anticipated.

## Abbreviations

COX: Cyclooxygenase
CV: Cardiovascular
FDA: United states food and drug administration
GI: Gastrointestinal
NSAID: Non-steroidal anti-inflammatory drug(s)
nsNSAID: Non-selective NSAID
PPI: Proton pump inhibitors
